# Incorporating Evidence-Based Parenting Practices into Home-Based Behavioral Health: A PCIT-Informed Approach for Training Paraprofessionals

**DOI:** 10.3390/children13020259

**Published:** 2026-02-12

**Authors:** Ashley T. Scudder, Jake C. Steggerda, Kathleen Clancy, Beatriz Mendez, Catherine Wright, Cheryl B. McNeil

**Affiliations:** 1 Partnerships in Prevention Science Institute, Department of Human Development and Family Studies, Iowa State University, Ames, IA 50010, USA; kclancy@iastate.edu; 2 Department of Psychiatry, University of Florida, Gainesville, FL 32610-0256, USA; j.steggerda@ufl.edu (J.C.S.); beatrizmendez@ufl.edu (B.M.); cheryl.mcneil@ufl.edu (C.B.M.); 3 Minnesota Department of Human Services, Saint Paul, MN 55164-09981, USA; catherine.wright@state.mn.us

**Keywords:** behavioral parent training, paraprofessionals, home-based services, Parent–Child Interaction Therapy

## Abstract

**Highlights:**

**What are the main findings?**

- Paraprofessional-delivered, PCIT-informed Child-Directed Interaction (CDI) skills practice was associated with large, clinically meaningful improvements in observed caregiver parenting behaviors within a brief intervention window.
- Caregiver-reported child disruptive behaviors decreased significantly during early Behavioral Skills Training for Families (BSF) program, indicating early behavior change in real-world, home-based services.

**What are the implications of the main findings?**

- Structured, PCIT-informed parenting skills can be effectively delivered by bachelor’s-level workers under supervision, expanding access to evidence-based practices in community settings.
- Behavioral Skills Training for Families (BSF) shows promise as a scalable workforce model that preserves core behavioral mechanisms while reducing common barriers to traditional clinic-based parent training.

**Abstract:**

Background/Objectives: Disruptive behavior problems are common in early childhood, yet access to evidence-based parent training remains limited in many communities due to workforce shortages and service delivery barriers. Behavioral Skills Training for Families (BSF) is a Parent–Child Interaction Therapy (PCIT)-informed, home-based behavioral skills practice model designed to be delivered by bachelor’s-level paraprofessionals under close supervision. This pilot study evaluated the feasibility and preliminary caregiver and child outcomes associated with the Child-Directed Interaction (CDI) module of BSF to inform refinement of training and implementation protocols and guide future evaluation. Methods: Using a non-randomized pre–post design embedded within routine services, caregiver–child dyads (children ages 2–10 years) receiving BSF CDI across community-based agencies in Minnesota were included. Outcomes were assessed using observational coding of caregiver skills (Dyadic Parent–Child Interaction Coding System; DPICS) and caregiver-reported child behavior measures (Eyberg Child Behavior Inventory [ECBI]; Weekly Assessment of Child Behavior–Positive [WACB-P]). Paired-sample *t*-tests with intent-to-treat analyses examined changes from the baseline to the last attended CDI session. Results: Caregivers demonstrated statistically significant and large increases in observed positive parenting skills and reductions in negative verbalizations during child-led play. Children showed significant reductions in disruptive behavior intensity and problem scores on the ECBI, reflecting movement toward clinically meaningful improvement. No significant change was observed in caregiver-reported positive child behaviors on the WACB-P. Post hoc analyses were conducted to further explore these differences and found consistent changes in the ECBI for cases, regardless of no reported changes in positive child behaviors on the WACB. Conclusions: The results provide preliminary evidence that a structured, PCIT-informed CDI skills practice model can be feasibly implemented by paraprofessionals and is associated with meaningful improvements in caregiver behavior and child behavior outcomes in the first 2–3 months following service initiation. The findings support BSF as a promising workforce-embedded approach and inform future controlled studies examining effectiveness, sustainability, and broader implementation outcomes.

## 1. Introduction

Disruptive behavior disorders remain among the most prevalent mental health conditions in childhood, affecting about 7% of children and adolescents in the United States [1,2]. Moreover, disruptive behavior difficulties commonly co-occur with other childhood conditions, such as attention-deficit/hyperactivity disorder (ADHD), autism spectrum disorder (ASD), various internalizing difficulties (e.g., depression and anxiety), and traumatic stress, often exacerbating these conditions and increasing the risk for more severe problems [3,4,5,6,7,8]. The presence of disruptive behavior problems has been consistently implicated in a host of adverse outcomes for youth across social, emotional, and academic domains of functioning [9,10,11]. Studies linking disruptive behaviors to greater caregiver stress, family conflict, and emotional/behavioral difficulties in siblings highlight the broader negative impact of disruptive behavior problems, as does research estimating their considerable economic and societal costs [12,13,14,15,16]. Given the massive harm associated with disruptive behavior problems for children, families, and society, there is a high need for treatment and prevention.

The need to treat and prevent disruptive behavior problems in children and their families has been addressed through a range of interventions and service models. For example, in Minnesota, commonly utilized evidence-based family interventions include Parent–Child Interaction Therapy (PCIT), which focuses on strengthening caregiver–child relationships and improving behavior through structured coaching [17]. School-based interventions are also frequently employed; for example, PracticeWise modular approaches are often implemented within educational settings to address behavioral concerns, though these services may not consistently involve caregivers or family systems. Within schools, children with disruptive behaviors may additionally receive targeted supports or accommodations, such as behavior intervention plans, to promote functioning and engagement [18].

Minnesota also has a robust rehabilitative children’s mental health service array through Children’s Therapeutic Services and Supports (CTSS), which frequently includes home-based, skills-focused interventions delivered by mental health practitioners or skills workers [19]. However, these providers often receive limited training in specific evidence-based practices for disruptive behavior, despite routinely working with children and families in naturalistic settings. This service context has generated interest in skills-based adaptations of PCIT that may align well with CTSS service delivery models and enhance the quality and consistency of interventions provided in the home. To further support children with complex emotional and behavioral needs, these interventions may be coordinated through wraparound care, an individualized, family-driven approach emphasizing collaboration and comprehensive service planning [20].

Despite significant support for the effectiveness of therapeutic interventions in reducing disruptive behaviors and facilitating other positive outcomes for children, the treatment programs, in their conventional form (e.g., clinic based and time intensive), are not always accessible to families in need, underscoring the need for more accessible evidence-based alternatives for children with disruptive behavior problems [21,22]. At-home services have the potential to offer an avenue to intervention for families facing these and other barriers preventing them from accessing traditional therapeutic care (e.g., cost of services, transportation, provider shortage, and time constraints) [23,24]. However, efforts to adequately address these barriers and deliver quality intervention to children at home can fall short when evidence-based practices are lacking or implemented inconsistently and when there is a shortage of qualified providers. For a review of home-based childcare services, see Porter et al. (2010) [25].

When it comes to at-home services, the tiered model implemented in ABA therapy stands out as an empirically supported intervention targeting disruptive behaviors. In ABA, a bachelor’s-level registered behavior technician (RBT) trained and certified in ABA techniques works directly with children in the home while under the supervision of a master’s-level board-certified behavior analyst (BCBA) [26]. In addition to creating and modifying the treatment plan the RBT follows, the BCBA provides ongoing training and support to the RBT and, crucially, ensures fidelity to the evidence-based practices of structured teaching and reinforcement employed in ABA [26]. While treatment under this model has yielded positive results, access to ABA services is often restricted to youth diagnosed with ASD [22]. Even among this group, access to care can be further limited by obstacles such as long waitlists and insufficient provider or service availability, leaving many children with disruptive behavior difficulties without adequate options for at-home interventions [27].

There is some available research supporting home-based intervention services in the context of, and as a supplement to, evidence-based parent training. For instance, in a randomized controlled trial, Lees and colleagues (2019) found greater improvements in child behavior over time for caregivers receiving a home-based parent support intervention (i.e., 10 h long sessions of personalized coaching with clinicians) relative to those participating in parent training alone (i.e., the Incredible Years program) [28]. Though such research speaks to the positive potential of home-based interventions to reduce behavior problems in youth, having clinicians trained in evidence-based techniques come to the home to deliver care is not always feasible. In Minnesota, a significant proportion of at-home mental health services for children are delivered through the Medicaid Children’s Therapeutic Services and Supports (CTSS) benefit, which emphasizes rehabilitative, skills-based interventions provided in naturalistic settings by paraprofessionals (i.e., mental health skills workers). Similar to other at-home services outside of ABA (e.g., respite care), these providers’ primary roles often focus on monitoring, support, and skills practice rather than delivering structured interventions. Accordingly, paraprofessional workers are typically trained in fundamental safety and caregiving skills and not necessarily in the evidence-based practices most directly associated with behavior change [29].

Parent–Child Interaction Therapy (PCIT), an intervention for early childhood disruptive behavior disorders, has a large body of empirical support. PCIT is a dyadic, behavioral parent-training intervention designed for children ages 2 to 7 years that targets disruptive behavior through live coaching of caregivers during structured parent–child interactions [18,30]. PCIT can be delivered either in clinic or via telehealth by master’s-level clinicians or above and consists of two phases: Child-Directed Interaction (CDI), which focuses on parent–child relationship enhancement through differential attention and positive interaction skills, and Parent-Directed Interaction (PDI), which emphasizes consistent limit setting and effective boundaries and limit-setting strategies. Past research consistently demonstrates that PCIT produces statistically and clinically significant reductions in child disruptive behaviors, increases in parenting skills acquisition, and decreases in caregiver stress [31].

Despite its strong evidence base, engagement in traditional PCIT is not feasible for all families. PCIT is resource intensive, requiring frequent sessions over an extended period, specialized clinic infrastructure (e.g., observation rooms and bug-in-the-ear coaching systems) if conducted in clinic, and access to clinicians with advanced training and certification [32]. Additionally, PCIT uses a competency model for treatment progression, meaning that families must meet certain skill criteria to progress through and graduate from treatment [30]. PCIT treatment criteria lend themselves to producing strong treatment effects but can be challenging for some families to meet quickly. These requirements can create barriers related to cost, transportation, scheduling constraints, workforce shortages, and geographic access, particularly for families living in rural or underserved areas [33,34]. Though families demonstrate benefit even from receiving some PCIT intervention without reaching graduation criteria, high attrition rates in PCIT highlight the challenge of sustaining engagement across the full course of treatment for many families [35,36].

Concerns about service dosage and efficiency have also prompted considerations of adapting PCIT to a fixed number of fewer sessions in order to balance family benefits and needs with a lower-intensity yet effective intervention. Study designs incorporating fixed-number sessions such as Scudder and colleagues (2019) demonstrate that meaningful improvements in parenting skills and child behavior can be demonstrated following a fixed number of CDI sessions for children with autism spectrum disorder (e.g., eight CDI sessions; ASD) [37]. These findings further demonstrated statistical and clinically significant improvements in disruptive behavior problems following 1 h of weekly PCIT even for children already receiving at least 16 h of other services.

Behavioral Skills Training for Families (BSF) was developed in response to the barriers noted above as a PCIT-informed, in-home behavioral skills practice model delivered by bachelor’s-level paraprofessionals (or behavioral skills workers). BSF draws heavily from PCIT, particularly in its focus on relationship enhancement and limit setting, but differs fundamentally in its implementation and clinical role. BSF is not considered a therapeutic intervention. Instead, it is a structured behavioral skills practice program designed to increase caregiver use of evidence-informed parenting strategies inside families’ homes.

In addition to its roots in PCIT, BSF is informed by Intensive Family Coaching (IFC), which is an earlier PCIT-informed home-based intervention model designed to support families who could not engage in traditional PCIT [38]. IFC demonstrated that direct delivery through a team model of a master’s-level therapist and paraprofessional, could effectively support caregiver skill acquisition and improve child behavior outcomes using intervention strategies informed by PCIT [38]. However, IFC retained therapist-led coaching and case complexity that limits its scalability to master’s-led therapist sessions. Notably, IFC requires both the master’s-level (or above) supervisor and bachelor’s-level paraprofessional to attend each session [38]. BSF builds upon these interventions by further defining the role of the paraprofessional as a skills worker focused on guided practice and structured repetition of parenting behaviors, rather than providing live coaching or handling in-the-moment clinical decision making.

One of the largest distinguishing factors between PCIT, IFC, and BSF is BSF’s omission of live coaching. Whereas PCIT and IFC therapists actively coach caregivers through parent–child interactions to shape behavior in real time, BSF emphasizes structured and prescribed brief skills practice. This distinction reflects both ethical and practical considerations. Coaching during elevated child dysregulation, particularly during extinction bursts that may occur when new behavioral contingencies are introduced, requires advanced clinical judgment and crisis management skills typically beyond the scope of paraprofessional training. A side-by-side comparison of PCIT, IFC, and BSF is presented in Table 1.

The BSF service delivery model shares structural similarities with the tiered workforce model described above in ABA intervention, particularly the use of bachelor’s-level providers delivering direct services under close supervision. In ABA, registered behavior technicians (RBTs) implement treatment protocols developed and overseen by board-certified behavior analysts (BCBAs), with an emphasis on fidelity monitoring and skill acquisition [26]. Typically, RBTs largely implement the intervention independently in home with patients with routine supervision from a BCBA. BSF similarly relies on intensive supervision and structured protocols to support skills workers’ implementation of evidence-informed practices.

However, BSF differs from ABA in several important ways. First, BSF is transdiagnostic and not limited to children with autism spectrum disorder, which serves to address a broader population of children with disruptive behavior difficulties. Second, BSF prioritizes caregiver skill development and dyadic interaction quality rather than child-only behavior modification, which is common with ABA. Finally, BSF aligns with state-level guidelines for bachelor’s-level behavioral skills workers, such as those used in Minnesota home-based services, which emphasize structured skill building, supervision, and safety rather than independent clinical decision making [39].

Training requirements further distinguish BSF from PCIT. Whereas PCIT clinicians typically complete 40–56 h of workshop training, followed by supervised cases and a year of consultation, BSF skills workers receive substantially less direct expert training focused specifically on CDI and Adult-Directed Interaction (ADI) skills. In the current implementation, skills workers complete approximately 16 h of remote training for CDI and 16 h for ADI, while supervisors complete additional preparatory training (i.e., 16–32 h). BSF supervisors also engage in regular consultation calls with the BSF development team. Notably, BSF compensates for reduced initial training with increased supervision frequency and fidelity monitoring through case tracking and ongoing consultation. Detailed training and supervision procedures are described in Section 2.

### Current Study

Given the evidence that meaningful changes in caregivers’ skills and childhood disruptive behaviors can be produced with several treatment sessions focused on relationship enhancement and selective attention (i.e., CDI skills) and evidence supporting the effectiveness of PCIT skill implementation in community settings, this study evaluates the potential effectiveness of the CDI phase of BSF delivered by bachelor’s-level behavioral skills workers [35,40]. Consistent with PCIT theory and prior empirical findings from PCIT and IFC interventions, we hypothesized that caregiver use of positive “Do” skills (e.g., labeled praise, reflections, and behavior descriptions) as measured by the Dyadic Parent–Child Interaction Coding System (DPICS) would increase, while use of negative “Don’t” skills (e.g., commands, questions, and criticisms) would decrease across BSF CDI-focused skills sessions [41]. We also expected reductions in child disruptive behaviors over the course of families’ involvement in the CDI phase.

Notably, this study is a preliminary look at BSF as a service delivery model and is intended to be exploratory in nature. Because of this, we focused our study exclusively on the CDI phase of BSF to evaluate whether change is possible in key intervention indicators traditionally used in PCIT. Evaluation of the Adult-Directed Interaction (ADI) phase, which focuses on limit setting and compliance, will be addressed in future research. By examining whether skills worker-delivered, PCIT-informed CDI skills practice can produce meaningful behavior change, this study aims to inform scalable, accessible service models for families who may not otherwise access traditional clinic-based interventions.

## 2. Materials and Methods

### 2.1. Study Design and Reporting Standards

This study utilized a non-randomized pre–post design with an intent-to-treat (ITT) analytic approach, focusing on families engaged in the Child-Directed Interaction (CDI) skills practice component of the Behavioral Skills Training for Families (BSF) program. All eligible families who initiated BSF CDI as part of routine services were included. This pilot study was designed to inform refinement of BSF training and implementation protocols and to establish feasibility and signal of change to guide subsequent, more rigorous evaluations. Because this was a single-condition pilot embedded within routine services, no assignment procedures or comparison groups were used. Additionally, CDI duration was skill based rather than session count based. Reporting adheres to the Transparent Reporting of Evaluations with Non-Randomized Designs (TREND) guidelines.

### 2.2. Conceptual Frameworks

Three complementary implementation science frameworks—the Consolidated Framework for Implementation Research (CFIR), the Practical, Robust Implementation and Sustainability Model (PRISM), and the RE-AIM Framework—guided decision making during the development/implementation of BSF and informed interpretation of pilot findings [42,43,44,45,46,47]. Rather than serving as a formal test of these frameworks, they were used pragmatically to shape early implementation choices, ensure contextual fit, and establish a foundation for more rigorous evaluation in future research.

CFIR provided a structured, theory-informed lens to consider multilevel factors likely to influence BSF implementation, including intervention characteristics, organizational context, workforce capacity, and implementation processes. These domains guided decisions related to training content, supervision structure, and adaptation of BSF delivery to agency contexts, helping to anticipate barriers and facilitators to early uptake.

PRISM complemented CFIR by emphasizing how intervention design features interact with organizational infrastructure, the external service environment, and implementation processes over time. This perspective informed protocol decisions aimed at supporting real-world feasibility and sustainability, including alignment of BSF with existing service workflows, workforce roles, and agency capacity. Whereas CFIR supported identification of key determinants, PRISM helped orient the project toward how these factors jointly shape implementation quality and longer-term maintenance.

The RE-AIM Framework provided an organizing structure for considering the potential public health impact of BSF during this pilot phase, with particular attention to reach, adoption, and implementation. RE-AIM guided early decisions about who the training was designed to serve, how BSF was delivered across settings, and what implementation indicators would be most informative at this stage. Together, these frameworks supported a developmentally appropriate, implementation-informed pilot approach that both strengthened the current training and implementation protocols and positioned the project for more comprehensive, theory-driven evaluation in subsequent studies.

### 2.3. Participants and Setting

Caregiver–child dyads (children ages 2–10 years) enrolled in BSF between May 2024 to December 2025 were eligible if they initiated BSF and completed baseline assessment and had at least one more data point after baseline the last available observation (Last Observation Carried Forward; LOCF). No additional exclusion criteria were applied. BSF was delivered in community-based agencies across Minnesota. Families were referred to BSF through standard agency intake and care coordination processes, including child welfare, mental health, and community referrals. Enrollment reflected standard service access rather than research recruitment. Thus, all participants received BSF as part of routine service delivery, and data were derived from program evaluation records collected during standard care.

#### 2.3.1. Child Characteristics

Children in the sample had a mean age of 5.34 years (*SD* = 2.19). Half of the sample was male (50%), with 19% female; gender was not reported for 31% of participants. The majority of children were identified as White (72%), with smaller proportions identifying as African American (2%), American Indian/Alaska Native (2%), or biracial (2%). Nearly half of the sample had an autism diagnosis (42%), followed by trauma- and stress-related disorders (16%), ADHD (8%), generalized or other anxiety disorders (6%), and other neurodevelopmental disorders (4%, e.g., intellectual disability). One child (2%) had no diagnosis, and for about 20% of the sample, no diagnostic information was reported. Comorbidity was common, with 14% of children diagnosed with two or more diagnoses. Regarding insurance coverage, 56% of children were insured through public insurance and 27% through private insurance. Baseline Child Behavior Checklist (CBCL) scores indicated clinically elevated symptom levels, with mean T-scores of 60.64 (*SD* = 12.54) for Internalizing Problems, 72.82 (*SD* = 11.02) for Externalizing Problems, and 68.27 (*SD* = 8.63) for Total Problems. Sixteen percent of families reported prior PCIT involvement. Family risk indicators included histories of corrections involvement (17%) and substance use (11%). In most cases, the primary caregiver lived with the child (73%) (see Table 2).

#### 2.3.2. Caregiver Characteristics

Caregivers participating in the study were predominantly mothers (60%), with fathers comprising 15% of the sample, and other caregivers accounting for 4%; caregiver role was not reported for 21% of participants. Most caregivers identified as White (81%), with 2% identifying as American Indian/Alaska Native; race/ethnicity was not reported by 17% of the sample. Regarding disability or mental health history, 17% of caregivers reported a history of disability or mental health concerns, 27% reported no such history, and 56% did not report this information.

#### 2.3.3. Workforce Characteristics

The BSF workforce included 8 supervisors and 58 frontline workers. Among those delivering BSF services, the majority provided services in person (93%), with a small proportion using a hybrid delivery format (7%). On average, workers carried about 2 cases, with most workers having at least 1 case (37%) and a range of 1–14 cases. For workers included in the sample, nearly half delivered sessions weekly (48%), followed by weekly to bi-weekly sessions (25%). For smaller proportions of cases, sessions delivered ranged from twice weekly (4%), weekly (12%), and bi-weekly (8%) to monthly. Following training, 15 participants (32%) dropped out or transitioned out of BSF service delivery. The most common reason for attrition was leaving the agency, followed by transitions related to clinical training or licensure status (*n* = 3). Less frequently reported reasons included finding BSF not helpful for service delivery (*n* = 2), role changes due to limited agency capacity (*n* = 1), and promotion (*n* = 1).

### 2.4. BSF Training and Intervention Delivery

#### 2.4.1. BSF Initiative and Skills Worker Training

BSF is a PCIT-informed, home-based behavioral skills practice initiative designed to support caregiver acquisition of evidence-informed parenting skills through structured practice delivered by bachelor’s-level behavioral skills workers under close supervision. The BSF initiative includes standardized training, guided session protocols, and ongoing consultation to support consistent implementation across community agencies.

Skills workers completed a structured training sequence focused on Child-Directed Interaction (CDI) and Adult-Directed Interaction (ADI) skills, including didactic instruction, modeling, guided practice, fidelity expectations, and case-based supervision. Training components were intentionally designed to address key implementation determinants, including intervention characteristics (e.g., structured skill sequences and fidelity tools), inner setting and workforce capacity (e.g., supervision models and role clarity), and implementation processes (e.g., standardized workflows and feedback loops), consistent with CFIR domains and PRISM’s emphasis on intervention–context fit and sustainability.

**Figure 1:**
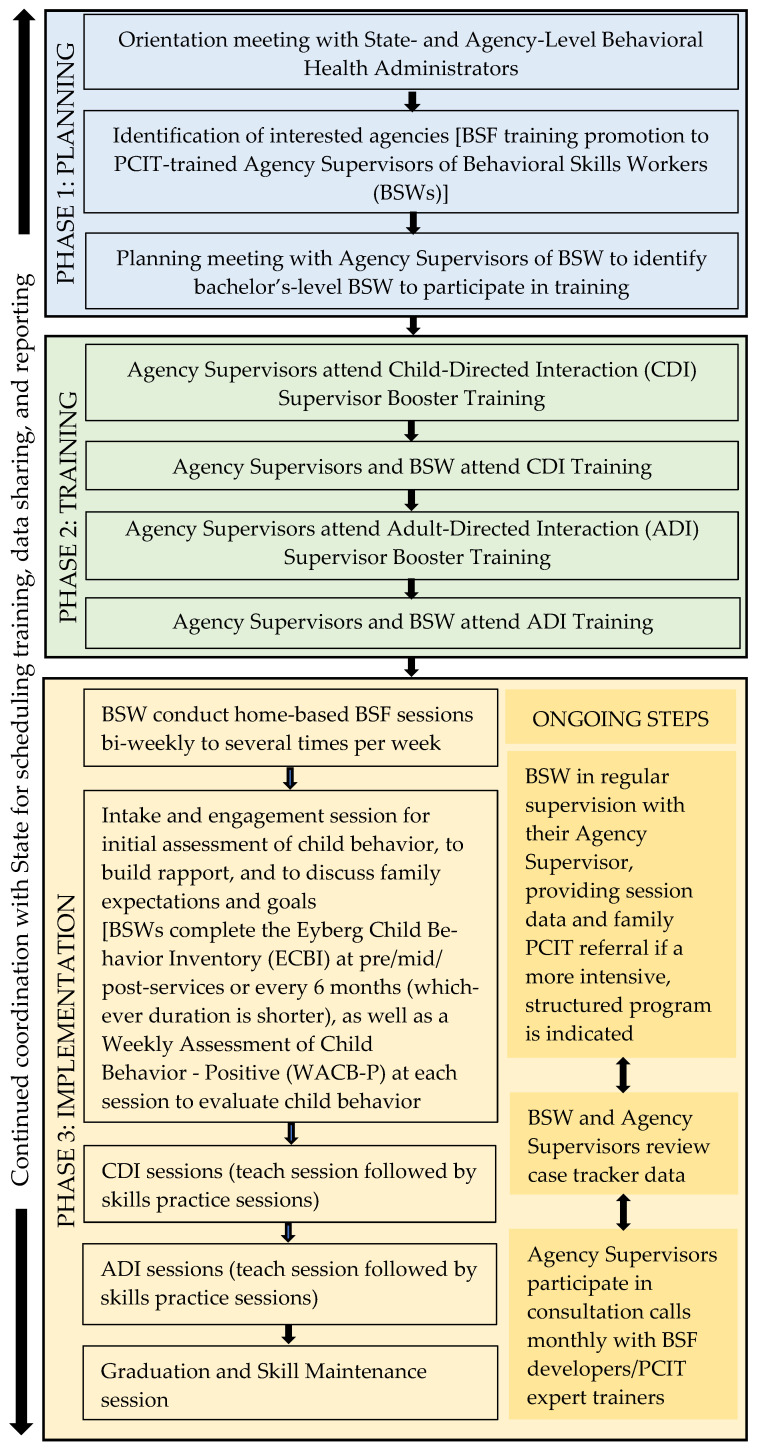
Behavioral Skills Training for Families (BSF) training process.

A comparison of BSF with Parent-Child Interaction Therapy (PCIT) and Intensive Family Coaching (IFC) is presented in Table 1, and an overview of the BSF training and implementation workflow is provided in Figure 1. Training materials and skills session guides are available from the corresponding author upon reasonable request. The description of training content, supervision structure, and implementation procedures provided here is intended to offer sufficient detail to enable replication and adaptation of the BSF training and service delivery model in other community-based settings.

#### 2.4.2. BSF Service Delivery: Child-Directed Interaction Module

The Child-Directed Interaction (CDI) skills practice module of BSF consists of weekly to bi-weekly structured skills practice sessions delivered in families’ homes by trained paraprofessional behavioral health skills workers. Each session follows a standardized protocol that includes a brief review of prior practice, skills instruction and modeling, guided in-session practice of CDI skills (e.g., labeled praise, reflections, and behavior descriptions), collaborative homework planning, and completion of structured fidelity and skills tracking tools. CDI length was designed to be determined based on caregiver skill level, with session frequency determined by agency scheduling and family availability.

Consistent with CFIR domains related to intervention characteristics and implementation processes and PRISM constructs emphasizing alignment with service workflows and feasibility, the CDI delivery protocol was intentionally designed to be highly structured, repeatable, and well matched to paraprofessional scope of practice while preserving the core relational mechanisms of PCIT-informed CDI.

The session structure, practice sequence, and fidelity procedures were supported by standardized session guides, caregiver homework planning, and skills tracking forms used across agencies in the current initiative.

### 2.5. Outcome Measures

#### 2.5.1. Caregiver Skill Change Outcomes

Caregiver behavior during play was assessed using the Dyadic Parent–Child Interaction Coding System (DPICS), a standardized observational coding system widely used in parent–child interaction research and clinical practice. DPICS captures the frequency of caregiver positive attention skills (e.g., labeled praise, reflections, and behavior descriptions) and reductions in negative behaviors (e.g., commands, questions, and criticisms) during structured child-led play. Skills workers completed DPICS observations at baseline and at each CDI skills practice session. DPICS observations were conducted during standardized child-led play segments and coded according to published DPICS-IV procedures [41].

#### 2.5.2. Child Behavioral Outcomes

Child behavioral functioning was assessed using caregiver report measures including the Eyberg Child Behavior Inventory (ECBI) and the Weekly Assessment of Child Behavior-Positive (WACB-P). All caregiver report measures were administered as part of routine BSF program monitoring and completed electronically or by being read aloud when necessary.

- Eyberg Child Behavior Inventory (ECBI) [47]:
  The ECBI Intensity and Problem Scales were completed at baseline and at completion of CDI component to assess caregiver-perceived child disruptive behavior. Psychometric studies indicate that ECBI scores in ASD samples reflect multiple behavioral dimensions—including emotional reactivity, attentional regulation, and noncompliance—rather than a unitary oppositional construct. Accordingly, in the present study, changes in ECBI scores were interpreted as indicators of early improvement in caregiver-perceived child behavioral regulation and family functioning [48].
- Weekly Assessment of Child Behavior–Positive (WACB-P) [49]:
  Caregivers completed the WACB-P at baseline and at each CDI session to assess short-term changes in child prosocial, regulated, and cooperative behaviors during the intervention period.

### 2.6. Data Collection and Participant Flow

**Figure 2:**
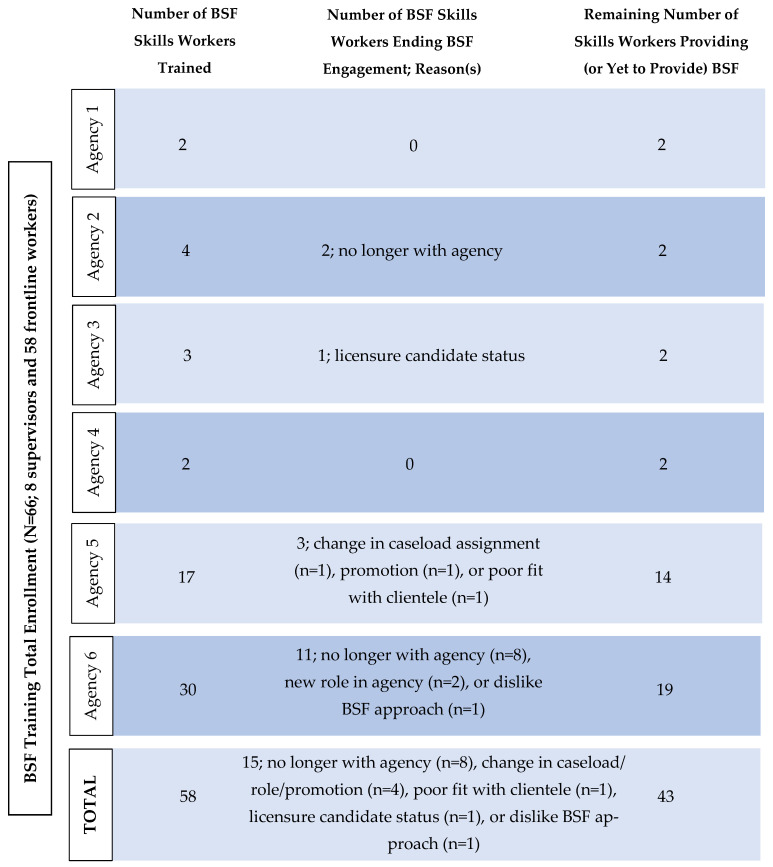
BSF participant flow.

Baseline assessments were completed before Session 1. Timepoint 2 data were collected during their last attended BSF CDI session to date (*M* = 7.10 sessions, *SD* = 3.94). Participant flow including enrollment, retention, and reasons for training discontinuation is outlined in a flow diagram (see Figure 2). Because BSF was delivered as routine care, formal screening logs were not maintained; all families who initiated BSF were considered eligible. Recruitment and data collection occurred between May 2024 and December 2025.

### 2.7. Analytic Approach

All analyses were conducted using R version 4.2.2 [50]. Descriptive statistics and demographic data are presented in Table 2. For our primary analyses, we conducted paired-sample *t*-tests using participants’ first and last reported CDI sessions. Specifically, we examined pre–post changes for parenting skills (i.e., DPICS “Do” and “Don’t” skills), child disruptive behaviors and child positive behaviors (i.e., ECBI Intensity, ECBI Problem, and WACB-P Intensity scores). Confidence intervals were not emphasized due to the pilot nature of the study and small sample sizes; effect sizes were prioritized to estimate the magnitude of change. For all *t*-test analyses, we computed Cohen’s *d* effect sizes, where *d* = 0.20, 0.50, and 0.80 represent small, medium, and large effect sizes, respectively) [51].

We conducted additional post hoc paired-sample *t*-test analyses with a subset of our sample for whom data were simultaneously available on the ECBI (*n* = 10 for Intensity; *n* = 8 for Problem), WACB-P (*n* = 10), and DPICS “Do” (*n* = 10) and “Don’t” (*n* = 9) skills at the initial and last CDI sessions. Analytic decisions were selected to balance rigor with feasibility for a pilot evaluation embedded within routine services. Analyses focused on within-subject change to identify signal of improvement and inform hypotheses for future controlled studies. Participating skills workers were not uninformed to intervention exposure due to the nature of service delivery.

#### 2.7.1. Intent to Treat (ITT)

All caregivers with baseline data were included in ITT analyses. For families who did not complete at least six CDI sessions, the last available observation (LOCF) was used as the Timepoint 2 data point.

#### 2.7.2. Pre–Post Change Analyses

Within-subject changes from baseline to Timepoint 2 were analyzed using paired *t*-tests:

- DPICS: Positive skill increase and negative skill reduction.
- ECBI: Intensity and Problem Scales.
- WACB-P: Prosocial and regulated behavior indicators.

Effect sizes (Cohen’s *d*) were calculated.

### 2.8. Generative Artificial Intelligence Use Statement

No generative AI tools were used to generate data, conduct analyses, interpret results, or create figures or tables. All final content was reviewed and approved by the authors, who take full responsibility for the manuscript.

## 3. Results

To test the extent to which children in BSF or their caregivers experienced change over the course of BSF CDI, we conducted a series of paired-sample *t*-tests. BSF supervisors reported ECBI data for 19 BSF families, WACB-P data for 33 families, DPICS “Do” skills for 47 families, and DPICS “Don’t” skills for 38 families. Patients (*n* = 48) completed a mean of 7.10 CDI sessions (*SD* = 3.94). A breakdown of participant characteristics (i.e., diagnoses, demographics, etc., can be found in Table 2). No adverse events or unintended negative effects related to BSF participation were reported during the study period.

### 3.1. Caregiver Skill Change

Paired-sample *t*-tests indicated that caregivers engaged in significantly more DPICS “Do” skills at their child’s last CDI session (*M* = 26.23, *SD* = 10.89, min = 5, max = 52) compared to their initial CDI session (*M* = 10.13, *SD* = 7.69, min = 0, max = 31; *t*(47) = 9.07, *p* < 0.001; Cohen’s *d* = 1.31). Similarly, caregivers engaged in significantly fewer “Don’t” skills at their child’s last CDI session (*M* = 3.23, *SD* = 4.75, min = 0, max = 21) compared to their initial CDI session (*M* = 14.07, *SD* = 12.00, min = 1, max = 46; *t*(43) = −6.39, *p* < 0.001; Cohen’s *d* = −0.96).

### 3.2. Child Disruptive Behavior Change

#### 3.2.1. ECBI

Children’s disruptive behaviors as measured by the ECBI Intensity and Problem Scales demonstrated a similar pattern. Children’s ECBI Intensity scores at their last CDI session were significantly lower (*M* = 112.58, *SD* = 39.06, min = 50, max = 189) than their ECBI Intensity scores at their initial CDI session (*M* = 138.37, *SD* = 31.75, min = 84, max = 197; *t*(18) = −4.77, *p* < 0.001; Cohen’s *d* = −1.10). Relatedly, children’s ECBI Problems scores showed the same pattern at the end of CDI (*M* = 8.53, *SD* = 7.06, min = 0, max = 21) compared to their initial CDI session (*M* = 14.32, *SD* = 8.75, min = 3, max = 28; *t*(16) = −4.53, *p* < 0.001; Cohen’s *d* = −1.10).

#### 3.2.2. WACB-P

However, children’s WACB-P scores at their last BSF CDI session (*M* = 32.58, *SD* = 11.14, min = 11, max = 48) were not statistically different from scores at their initial BSF CDI session (*M* = 34.36, *SD* = 9.17, min = 13, max = 56; *t*(33) = −1.18, *p* = 0.248; Cohen’s *d* = −0.20).

### 3.3. Post Hoc Analyses

Post hoc analyses were exploratory and conducted to examine patterns among families with complete multi-measure data. Specifically, post hoc analyses were conducted examining change over time of a subset of families for whom data were concurrently available on the ECBI (*n* = 10 for Intensity; *n* = 8 for Problem), WACB-P (*n* = 10), and DPICS “Do” (*n* = 10) and “Don’t” (*n* = 9) skills at the initial and last CDI sessions (see Table 3).

#### Post Hoc Pre–Post Analysis

Paired-sample *t*-tests evaluating changes in disruptive behaviors as measured by the ECBI revealed significantly lower ECBI Intensity scores at the family’s last CDI session (*M* = 102, *SD* = 40.38, min = 50, max = 166) relative to their initial session (*M* = 130.20, *SD* = 33.21, min = 84, max = 187; *t*(9) = 4.62, *p* = 0.001, *d* = −0.72). Children’s ECBI Problem scores were also significantly lower at the last CDI session (*M* = 9.13, *SD* = 8.46, min = 0, max = 21) compared to their initial CDI session (*M* = 14.50, *SD* = 9.90, min = 3, max = 28; *t*(7) = 3.22, *p* = 0.014, *d* = −0.55). Paired-sample *t*-tests examining caregiver skills indicated significantly more DPICS “Do” skills in their child’s last CDI session (*M* = 31.70, *SD* = 8.31, min = 13, max = 41) relative to their initial BSF CDI session (*M* = 8.70, *SD* = 8.53, min = 0, max = 31; *t*(9) = −6.71, *p* < 0.001, *d* = 2.73). Caregivers additionally demonstrated significantly fewer DPICS “Don’t” skills in their last CDI session (*M* = 2.78, *SD* = 3.56, min = 0, max = 10) compared to their initial session (*M* = 16, *SD* = 10.67, min = 3, max = 35; *t*(8) = 3.77, *p* = 0.005, *d* = −1.58). By contrast, caregiver report of children’s positive behaviors as assessed by the WACB-P revealed that scores at the last CDI session (*M* = 31.70, *SD* = 11.71, min = 11, max = 46) did not differ significantly from those at the initial CDI session (*M* = 31.70, *SD* = 6.73, min = 22, max = 46; *t*(9) = 0.00, *p* = n.s., *d* = 0).

## 4. Discussion

### 4.1. Overview of Study Purpose and Key Findings

The present study evaluated short-term caregiver and child outcomes associated with the Child-Directed Interaction (CDI) skills practice module of Behavioral Skills Training for Families (BSF), a PCIT-informed, paraprofessional-delivered intervention implemented within home-based behavioral health services. Consistent with the study’s working hypotheses, the results indicated meaningful improvements in observed caregiver–child interaction quality alongside caregiver-reported gains in child prosocial and regulated behaviors during the first six BSF CDI sessions. These findings extend the evidence base for PCIT-informed approaches by demonstrating that core relational parenting skills can be effectively taught and practiced within a paraprofessional service delivery model, with measurable benefits emerging early in treatment. Taken together, these findings suggest that BSF functions not only as an early intervention strategy for families but also as a pragmatic workforce model that embeds evidence-based parenting mechanisms within existing home-based behavioral health systems.

### 4.2. Interpretation of Caregiver Skill Change

Observed increases in caregiver positive attention skills (e.g., labeled praise, reflections, and behavior descriptions) and concurrent reductions in directive or negative verbalizations during child-led play are consistent with prior research demonstrating that CDI skill acquisition is a primary mechanism of change in improving parent–child interaction quality [52]. Importantly, these changes were detected within a relatively brief intervention window and in a real-world service context, suggesting that paraprofessional skills workers can successfully support caregiver behavior change when training emphasizes structured practice.

Importantly, the magnitude of observed caregiver skill change suggests not only statistical significance but also meaningful clinical change. The large effect sizes observed for increases in DPICS “Do” skills (*d* = 1.31) and reductions in “Don’t” skills (*d* = −0.96) are comparable to effect sizes reported in early phases of clinician-delivered PCIT [31,53]. From a clinical standpoint, these changes reflect substantial shifts in caregiver interaction patterns toward increased positive attention and reduced directive or critical verbalizations, behaviors that are central mechanisms of change in PCIT and are associated with downstream improvements in child behavior and relationship quality. The degree of skill acquisition observed within a relatively brief CDI exposure suggests that BSF may support clinically meaningful caregiver behavior change even prior to completion of a full parenting intervention protocol.

These findings align with previous research indicating that early CDI skill gains often precede broader changes in child behavior and family functioning [53]. The use of DPICS observational coding strengthens confidence that changes reflect meaningful shifts in caregiver behavior rather than perception alone. From an implementation perspective, these results support the feasibility of embedding standardized observational assessment and skills practice strategies into community-based paraprofessional services to teach core behavioral parent-training strategies (e.g., differential attention, responsiveness, and reduced directive talk).

### 4.3. Child Behavioral Outcomes and Early Treatment Effects

Caregiver-reported improvements in ECBI Intensity and Problem scores suggest early improvements in child behavioral regulation during the CDI phase of BSF. These findings are consistent with prior work demonstrating that changes in caregiver responsiveness and attention during CDI are associated with reductions in disruptive behavior intensity and improvements in child regulation and compliance [54,55]. Reductions in ECBI scores also appear clinically meaningful. Mean decreases in ECBI Intensity exceeded thresholds commonly interpreted as reliable change, with large effect sizes (*d* ≈ −1.10). Given that baseline ECBI scores were in the clinically elevated range, these reductions suggest movement toward improved daily functioning and reduced caregiver burden. For 32% of children with available ECBI data, disruptive behavior improved to within-normal limits by the end of CDI, while an additional proportion demonstrated meaningful improvement despite remaining above clinical cutoffs, an expected pattern during early phases of PCIT-informed interventions prior to mastery or graduation [54,55].

Interpretation of ECBI change is particularly important given the diagnostic composition of the sample, in which nearly half of participating children had an autism spectrum disorder diagnosis. In ASD populations, ECBI scores reflect a multidimensional behavioral profile encompassing emotional reactivity, attentional regulation, and noncompliance rather than oppositional behavior alone. From this perspective, observed ECBI reductions likely reflect early improvements in emotional regulation and dyadic interaction quality—domains directly targeted during CDI—rather than broad resolution of disruptive behavior difficulties. These findings align with theory and prior empirical work suggesting that early CDI-focused interventions primarily reduce coercive interaction patterns and behavioral escalation, laying the groundwork for later gains in adaptive and prosocial functioning.

In contrast to ECBI findings, caregiver-reported positive child behaviors as measured by the WACB-P did not demonstrate statistically significant change during the CDI phase. This pattern likely reflects the timing and sensitivity of the measure rather than an absence of meaningful child change. Early CDI interventions are theorized to influence child behavior initially through reductions in dysregulation and disruptive behavior, with observable increases in positive or prosocial behaviors emerging later, particularly following the introduction of limit-setting components such as ADI or PDI [54,55]. Although prior research supports the sensitivity of the WACB-P to treatment-related change [49,56], the present findings suggest that it may be less responsive to early-phase BSF CDI change. The convergence of findings across the main and post hoc analyses—showing significant improvements in parenting skills and disruptive behavior but not positive behavior—further underscores this interpretation. Future research should examine the temporal sensitivity of positive behavior measures and consider combined use of complementary instruments (e.g., WACB-N and WACB-P or ECBI alongside positive behavior measures) to more fully capture both reductions in disruptive behavior and growth in adaptive functioning during different phases of intervention.

#### Clinical Significance and Prevention Implications

From a clinical perspective, these reductions are meaningful given that many children entered services with clinically elevated ECBI scores and multiple co-occurring risk factors. Notably, these improvements emerged early in treatment, with measurable reductions in disruptive behavior intensity observed within the first 2–3 months of service delivery. Early reductions in disruptive behavior intensity may reduce caregiver stress and interrupt coercive interaction cycles before escalation into more entrenched behavior patterns. Such early change is particularly relevant in home-based service settings, where prevention of worsening trajectories may be as critical as full symptom remission.

### 4.4. Implications for Paraprofessional Workforce Models

A central contribution of this study is its focus on paraprofessional-delivered parenting intervention within home-based behavioral health systems. Workforce shortages, particularly in rural and under-resourced communities, continue to limit access to evidence-based parenting interventions [57,58]. The current findings indicate that a structured BSF training model emphasizing CDI skills practice can be successfully implemented by paraprofessional staff under regular supervision, supporting both caregiver skill acquisition and early (i.e., within 2–3 months) child behavior change. Notably, the effect sizes observed in post hoc analyses were large across both caregiver skills and child disruptive behavior outcomes, suggesting that for families with complete multi-measure data, changes were not only statistically detectable but also likely to be observable and meaningful in daily family interactions.

Considering the Consolidated Framework for Implementation Research (CFIR), the BSF model appears to demonstrate strong intervention–context fit by aligning core PCIT-informed relational parenting principles with the realities of home-based behavioral health service delivery (CFIR). Specifically, BSF leverages existing paraprofessional roles, supervision structures, and visit schedules while introducing a highly structured, skills-based approach that emphasizes observable caregiver behaviors, repeated practice, and live feedback (PRISM) [44]. This alignment may reduce common implementation barriers associated with therapist-only models—such as limited-service capacity—while preserving fidelity to the active ingredients of evidence-based parenting interventions (CFIR) [42,43]. From a RE-AIM lens, the observed improvements in caregiver skills and early child behavior outcomes suggest promising effectiveness, while the use of paraprofessionals and integration within existing service systems supports adoption and potential for broad reach [45,46]. Taken together, these findings suggest that BSF may function not only as a paraprofessional-delivered intervention but also as a workforce development strategy that equips paraprofessionals with clearly defined behavioral competencies and coaching tools (CFIR and PRISM). By lowering training and delivery barriers without relaxing fidelity expectations, BSF may expand access to evidence-based parenting support across diverse service settings while maintaining adherence to core behavioral and relational mechanisms of change (CFIR and RE-AIM Maintenance).

Viewed through an implementation science lens, these findings align with CFIR domains related to intervention characteristics (e.g., structured protocols and observability of skills), individual characteristics (e.g., skills worker readiness for structured practice), and implementation processes (e.g., supervision and feedback loops). PRISM further highlights how BSF’s design aligns with organizational workflows and workforce sustainability by fitting within existing paraprofessional roles while minimizing reliance on scarce licensed clinicians. From a RE-AIM perspective, the ability to demonstrate early effectiveness within routine services supports the potential reach and adoption of BSF across diverse community settings, even when implementation is partial or evolving.

### 4.5. Limitations

Several limitations should be considered when interpreting these findings. First, the non-randomized pre–post design limits causal inference, and results should be interpreted as preliminary evidence of effectiveness rather than definitive treatment effects. Second, reliance on a modest sample size using the last observation carried forward (LOCF) for families who did not complete at least six CDI sessions may underestimate or overestimate true change for some participants. Third, the sample was drawn from a single statewide implementation context, which may limit generalizability to other service systems, populations or geographical locations.

Additionally, while the inclusion of observational DPICS data strengthens internal validity, child outcomes relied primarily on caregiver reports. Reliance on caregiver report measures may be influenced by expectancy effects, underscoring the value of complementary observational and multi-informant assessment strategies in future evaluations. Finally, the short follow-up window precludes conclusions about the durability of observed changes or their translation into longer-term child behavioral health outcomes.

### 4.6. Next Steps and Future Research Directions

Future research should prioritize randomized or quasi-experimental designs to more rigorously evaluate BSF effectiveness relative to usual care and master’s-level clinician-delivered models. Further, direct examination is needed of intervention dosage and dose–response relationships within BSF, including the number of CDI sessions needed to produce reliable reductions in disruptive behavior and subsequent gains in positive child functioning. Clarifying whether early ECBI improvements predict later adaptive behavior change will be critical for optimizing BSF duration, sequencing, and transitions between CDI and ADI phases. Additionally, examination of the full-course of BSF (i.e., including Adult-Directed Interaction) is also needed, as well as evaluations including longer follow-up periods to assess maintenance of caregiver skill use and downstream effects on critical outcomes such as child behavior, school functioning, and family stress.

Future research should also examine implementation processes that are critical to the scalability and sustainability of BSF, including predictors of paraprofessional skills, worker retention, and fidelity over time. Home-based behavioral health settings present unique challenges, as families often experience higher levels of environmental chaos and competing stressors that may interfere with consistency of skills practice and session structure. In addition, the BSF model represents a substantive shift from many paraprofessionals’ prior roles, requiring extended onboarding, longer intake processes, and early investment in supervision and modeling. It is possible that implementation efficiency and family engagement improve as organizational culture develops and as families are introduced to BSF as an initial service model rather than transitioning from more eclectic or less structured approaches. Future studies should test these hypotheses directly, including examination of implementation trajectories during early versus later phases of implementation.

Although the ECBI is well validated for use with children with ASD and other neurodevelopmental conditions, its multidimensional structure suggests that total score change may obscure differential improvement across behavioral domains (e.g., emotional reactivity versus attention difficulties). Future studies would benefit from examining factor-level ECBI change to further clarify mechanisms of improvement within paraprofessional-delivered models. Additional work should also examine implementation moderators, including paraprofessional training dose, intervention fidelity, and organizational supports that optimize outcomes. Finally, research is needed to explore adaptation and fit of BSF for skills workers working in additional service contexts, such as school-based services and early intervention systems, where role expectations, family engagement patterns, and organizational constraints may differ from home-based delivery.

Qualitative observations from the current initiative suggest that paraprofessional engagement with BSF varied, with a few skills workers expressing discomfort with the model. This resistance may reflect broader system-level norms favoring unstructured or eclectic service delivery rather than shortcomings of BSF itself. Consistent with CFIR constructs related to individual characteristics and implementation climate, transitioning from unstructured support roles to a highly structured, skills-based model may require additional onboarding, modeling, and reinforcement.

Additionally, workforce turnover and role transitions—common challenges in paraprofessional service systems—underscore the importance of designing implementation models that are resilient to staff changes and that prioritize rapid skill acquisition, clear role definition, and scalable supervision supports.

Given the growing interest in technology-enhanced interventions and measurement, future studies may also explore the integration of digital tools to support real-time feedback, observational coding, and caregiver engagement to support paraprofessional adoption of structured skills practice. Such approaches could further strengthen scalability while maintaining fidelity to behavioral parent-training principles.

## 5. Conclusions

This study provides preliminary evidence that a PCIT-informed, paraprofessional-delivered CDI skills practice module can produce meaningful improvements in caregiver–child interaction quality and early child behavioral outcomes within home-based behavioral health services. By demonstrating the feasibility and promise of BSF in real-world settings, these findings contribute to ongoing efforts to expand access to evidence-based parenting support through workforce innovation. Continued research is warranted to evaluate long-term outcomes, optimize implementation strategies, and clarify the role of paraprofessional models in reducing disparities in access to high-quality parenting interventions.

## Figures and Tables

**Table 1:** Treatment differences between Parent–Child Interaction Therapy (PCIT), Intensive Family Coaching (IFC), and Behavioral Skills Training for Families (BSF).

Intervention Component	PCIT	IFC	BSF
Training/Agency-Based Components			
Training	56 in-person hours delivered by a PCIT expert trainer	32 in-person hours delivered by a PCIT expert trainer	32 in-person or virtual hours delivered by a PCIT expert trainer, additional 32 h supervisor prep training
Post-training consultation	1 year, provided twice monthly by external consultant	Ongoing, as needed, provided by internal team	1 year, provided to BSF supervisors monthly by external consultant
Certification	Clinician level	Agency-level rostering	Provider-level rostering and supervisor-level rostering
Therapist/skills worker credentials	Licensed, master’s degree (minimum)	Master’s degree (minimum), mobile therapist qualification	Bachelor’s-level or equivalent behavioral skills worker, under master’s- or PhD-level supervision
Agency parameters	Outpatient setting	Connected to an ongoing, successful PCIT program	May be connected to an ongoing, successful PCIT program
Setting	Clinic based or telehealth	Home based	Home based
Service Delivery-Specific Components			
Siblings	Sibling may attend in final, skill-generalization session	Siblings may be present in home; simultaneously attended to by bachelor’s-level therapist	Siblings may be present in home; attended to by another family member
Technology	One-way mirror, earpiece, and microphone speaker system	Walkie-talkie and earpiece communication system	N/A
Staff needs	Single therapist	Therapist team (1 master’s level and 1 bachelor’s level)	Single bachelor’s-level behavioral skills worker receiving regular agency supervision
Average frequency of sessions	Once weekly	Twice weekly	Up to several times weekly
Duration of sessions	50–60 min	90–120 min	45–60 min
Average treatment length	12–20 sessions over six months; maintenance sessions not typically conducted	24–36 sessions over six to eight months; maintenance sessions may be conducted	Approximately 24 sessions; maintenance sessions may be conducted
Intervention phases	Child-directed interaction (CDI); parent-directed interaction (PDI; includes effective commands, time out)	CDI; adult-directed interaction (ADI; includes effective commands, hand-over-hand guide, and restriction of privilege)	CDI; ADI
Introduction to treatment	Conducted within CDI teaching session	Dedicated initial treatment engagement session; ongoing engagement/motivation discussion built into sessions	Dedicated intake/engagement session; ongoing engagement/motivation discussion built into sessions
Cultural awareness/tailoring	No systematic focus, ongoing cultural sensitivity/ consideration expected	Manualized focus with ongoing, collaborative discussion	Manualized focus with ongoing, collaborative discussion
Integration into services	Outpatient service	Integration into wraparound service prescription	Integration with outpatient PCIT or as standalone in-home service

Table adapted and reprinted with permission from Hybrid Implementation Effectiveness Trial: Home-based Intensive Family Coaching to Improve Outcomes for Medicaid-enrolled Preschoolers, Herschell, A. D., Shaffer, S. L., Wallace, N. M., Maise, A. A., Schaffner, K. F., McNeil, C. B., … Johnson, V. J. (2021), Evidence-Based Practice in Child and Adolescent Mental Health [38]. Copyright © 2021 Society of Clinical Child and Adolescent Psychology, reprinted by permission of Informa UK Limited, trading Taylor & Francis Group, https://www.tandfonline.com on behalf of Society of Clinical Child and Adolescent Psychology.

**Table 2:** Child demographics.

Characteristic	*M* (*SD*)	Percentage
Age		
Mean Age (in Years)	5.34 (2.19)	—
Gender		
Male	—	50%
Female	—	19%
Not Reported	—	31%
Race/Ethnicity		
White	—	72%
African American	—	2%
American Indian/Alaska Native	—	2%
Biracial	—	2%
Not Reported	—	22%
Diagnoses (Classification)		
Autism	—	42%
ADHD	—	8%
Trauma- and Stressor-Related Disorder	—	16%
Anxiety Disorder	—	6%
Oppositional Defiant Disorder (ODD)	—	2%
Other Neurodevelopmental Disorder	—	4%
No Diagnosis	—	2%
Not Reported	—	20%
Comorbidity		
One Diagnosis	—	61%
2 Diagnoses	—	5%
3 or more Diagnoses	—	9%
No diagnosis	—	2%
Not reported	—	23%
Insurance		
Private	—	27%
Public	—	56%
Hybrid	—	2%
Not Reported	—	15%
Baseline CBCL T-Scores		
Internalizing Problems ^a^	60.64 (12.54)	—
Externalizing Problems ^b^	72.82 (11.02)	—
Total Problems ^b^	68.27 (8.63)	—
PCIT Involvement		
Yes	—	16%
No	—	60%
Not Reported	—	24%
Family Corrections Involvement History		
Yes	—	17%
No	—	76%
Not Reported	—	7%
Family Substance Use History		
Yes	—	11%
No	—	63%
Not Reported	—	26%
Primary Caregiver Not Living with Child		
Yes	—	7%
No	—	73%
Other	—	2%
Not Reported	—	18%

^a^ At risk; ^b^ clinically significant.

**Table 3:** Post Hoc Pre–Post Analyses.

	Initial CDI Session		Last CDI Session			
	*M*	SD	*M*	SD	*t*	*d*
*Parenting Skills*						
DPICS “Do” Skills	8.70	8.53	31.70	8.31	−6.71 ***	2.73
DPICS “Don’t” Skills	16	10.67	2.78	3.56	3.77 **	−1.58
*ECBI Scores*						
Intensity	130.20	33.21	102	40.38	4.62 **	−0.72
Problem	14.50	9.90	9.13	8.46	3.22 ***	−0.55
*WACB-P Scores*						
Intensity	31.70	6.73	31.70	11.71	0.00	0.00

Note. ** *p* < 0.01, and *** *p* < 0.001.

## Data Availability

De-identified data are available from the corresponding author upon reasonable request, subject to agency data-sharing agreements and IRB constraints.

## Abbreviations

The following abbreviations are used in this manuscript:

BSF	Behavioral Skills Training for Families
PCIT	Parent–Child Interaction Therapy
WACB	Weekly Assessment of Behavior Change
CFIR	Consolidated Framework for Implementation Research
LOCF	Last Observation Carried Forward
